# On Paralysis of the Muscles of Inspiration

**Published:** 1838-01

**Authors:** 


### Art. VIII.

*Ueber Paralyse der Inspirations-Muskeln.* Von Dr. Louis Stromeyer. —*Hanover*, 1836.

*On Paralysis of the Muscles of Inspiration.* By Dr. Louis Stromeyer.—*Hanover*, 1836. 8vo. pp. 144.

In introducing Dr. Stromeyer's work to our readers, we shall, doubtless, recall to the recollection of many of them an intelligent young German who, ten years ago, was assiduously engaged in studying the practice of this country in the London hospitals. That he has not been unprofitably occupied since that time the present work is a sufficient evidence, as are also the better-known and successful efforts which he has made to extend and improve our knowledge of the nature and treatment of club-foot.

The present volume professedly treats of a very common disease, *Curvature of the Spine*, although it will not readily be recognized under the disguise of the new title in which it is here presented to the profession.

Minute investigation of the mechanism of inspiration has led Dr. Stromeyer to conclude, that the ordinary form of lateral incurvation is produced by paralysis of the respiratory nerves of the external muscles of inspiration. This is his fundamental proposition, the very groundwork on which all his researches are based; and he has, therefore, chosen to bring it prominently forward in his title-page, in order to attract attention to what he considers as almost alone important in the pathology of the disease. Whatever opinion may be formed of the general soundness of Dr. S.'s views, or of the extent of their application in the care and treatment of lateral curvature, no one who studies them will deny that they are at once novel and ingenious, and that they must lead to such a reconsideration of the whole subject as cannot fail to be highly beneficial, even if the author's own doctrines should fail to be adopted either wholly or in part. We ourselves by no means assent to all our author's views, and think that he has failed to prove, in a satisfactory manner, the very fundamental proposition of his system. Still, as the doctrines promulgated are of a very original character, are based on anatomy and physiology, and only framed after long and extensive observation of the morbid affections in all their forms, and as the author is a man of probity and great knowledge, and writes in the full conviction that what he advances is of high importance to the medical art, we deem it our duty, to lay before our readers an analytical epitome of the work. In doing so we shall, in the first instance, interpose but few critical comments of our own. At the conclusion of the article, we may perhaps introduce some observations on the general theory or on particular parts of it; but our professed object, on the present occasion, is rather to supply our readers with materials for reflection than to direct their judgment in one particular course. On considering for a moment the very extensive prevalence of lateral curvature of the spine, (or *Scoliosis*, as Dr. Stromeyer calls it, applying correctly the ancient term σκολιωσις) among the fashionably-educated young women of the present day, and the numerous evils, immediate and remote, of which it is the source, it is hardly possible to estimate too highly any doctrines which offer a prospect of preventing or curing it with greater certainty.

After devoting about thirty pages to the detail of cases, the author reminds us, that the inspiratory muscles possess two distinct and separate functions. The one pertaining to voluntary motion, the other to respiration. According to Sir Charles Bell, those different functions are performed through different sets of nerves. Therefore, in palsy of the respiratory nerves, the motive influence of volition is not necessarily withdrawn.

"The external inspiratory muscles, which have been hitherto believed to cooperate in the instance of accelerated and deep inspiration only, exert nevertheless a continued influence upon the dilatation of the chest. If the sterno-mastoid, trapezius, levator anguli scapulæ, rhomboidei, together with the serratus muscle of one side, no longer conduce to inspiration, in consequence of paralysis of their respiratory nerves, namely, of the accessorius and posterior thoracic; then are the scapula and ribs dragged downwards by the diaphragm, and the circumference of the corresponding side perceptibly lessened; in simultaneous paralysis of all the outward inspiratory muscles, the whole thorax becomes narrowed to the extent sometimes of five inches. It might seem doubtful whether the above-mentioned effect did not depend on the action of the intercostals alone; were it not demonstrable by experiment, that these muscles are of themselves incompetent to maintain the intermediate degree of thoracic dilatation. This dilatation is not the result of sensible contraction alternating with relaxation, but of prolonged organic tension: and is effected, by means of muscles subservient to the constantly shifting positions of the body, and to the incessant wants of respiration. In consequence of the extinction of this organic tension, the diaphragm is found to be much more elevated in the dead body than in expiration during life." (P. 34.)

In reference to the diaphragmatic retraction, there is a conclusive experiment related by Magendie. That physiologist laid open the costal pleura in a young animal, and marked the point to which the diaphragm rose. He then killed the animal by dividing the medulla oblongata, and observed the diaphragm to mount considerably higher. It is possible that a person during forcible expiration might elevate the diaphragm to much the same height as occurs in the dead body, but such an expiration can only be imagined in conjunction with complete relaxation of the inspiratory muscles. From these and subsequent remarks, the author seems to refer the phenomena in question to some power which maintains the due degree of balance and antagonism of the muscles, and firmness of the limbs; in other words, to that which Dr. M. Hall has termed the *reflex*, or complementary function of the nervous system.

Having enumerated the nerves which supply the principal external muscles of respiration, the author proceeds:

"Concerning the inspiratory nerves which supply the scaleni, the serratus posticus superior, the supra and infra spinatus, and the subscapularis muscle, we are still in the dark. It is to be hoped, however, that the increasing zeal of experimental physiologists will induce them to investigate the subject, now that its importance is so prominent." . . . "The configuration of the chest depends entirely on its normal dilatation. Interruption to the uniform respiratory action of both halves will lead sooner to deformity than interruption to the motive influence of volition; inasmuch, as the calls for respiratory action are unceasing, while voluntary motion is only occasionally brought into play." (P. 36.)

The author impugns Sir C. Bell's view of the mechanism of expiration, and adopts that of Borelli.*

"Of the respiratory motions inspiration is unquestionably the most important; requiring the greatest effort, it is essentially active; while expiration may be deemed the return to a state of repose. The elasticity and tone belonging to muscular texture suffices for the automatic movement of expiration. But for inspiration, a peculiar set of nerves is indispensable, denominated respiratory." (P. 41.)

In concluding this the introductory chapter, the author observes, that, "since nothing is known of the sympathies of the inspiratory muscles in disease, it is probable that various symptoms have been imputed to the lungs, which are alone explicable on the supposition of functional disturbance in these muscles. Where is the physiologist who has bethought himself of this principle for elucidating the phenomena of fever, of asthma, or of spinal distortion?"

The second chapter is an enquiry concerning the erect position of the human body and the lateral equilibrium of the trunk. The author's ideas on these matters are peculiarly deserving of notice. Although at first sight paradoxical, they nevertheless repose on principles of sound mechanics.

"In speculating as to the cause of the erect position, and the equilibrium of the trunk, most writers seem impressed with the belief, that the pelvis is the base or fixed point whence the muscles proceed to the spine, like the stays to the mast of a ship. In this way a part of the muscles of the spine certainly do operate. The mechanism for sustaining the column is however more complicated, and inconsistent with such a comparison. The head and cervical vertebræ constitute the basis whence the equilibrium of the chest along with the spinal column may be preserved. This is accomplished by means of the muscles of inspiration attached to the occiput and vertebræ of the neck; on the other hand, the expiratory muscles derive the great measure of their strength from the pelvis. To comprehend how the trunk of the body forms a

* "In placid and natural expiration the air is expelled from the lungs, not by the motive power of any muscles, but in virtue of the cessation of action in the intercostals and diaphragm, with simultaneous dilatation of the rima glottidis." *Borelli de Motu Animali.* Part II. prop. xcii. Hagæ, 1743.

strong but moveable shaft or radius, the head being the fixed point or centre, it will be convenient to contrast the arrangement of parts with a mechanism occasionally employed in the construction of bridges. It is thus that a long slender ladder or other similar object can be considerably strengthened by fastening to both ends cords firmly stretched and held apart at intervals by *straining-pieces;* an artifice resorted to in time of war, for mural-ladders required to bear the burthen of a number of men at one time; or for weak rafters laid across a stream, to supply a temporary bridge. The extremities of the ladder, or plank, constitute two fixed points whence the cords are stretched out, and by the equable distribution of the force of sustentation over the several points of disjunction, equilibrium is assured. The spine may be said to represent the weak ladder, the muscles the cords rendered tense by organic tension, and the alternate acts of inspiration and expiration; the strain being effected superiorly by the ribs and lungs, inferiorly by the abdominal viscera and their gaseous contents. According to this view, the intermediate state of activity of the collective muscles of the trunk upholds the erect position, and at the same time keeps the shoulder and ribs at their natural elevation. Towards inspiratory motion, the having a fixed point in the head is requisite; indeed the nature of the costal articulations upon which hinges the elevation of the thorax implies as much, because the chest does not advance upon the first rib as was formerly asserted, but upon the head and cervical vertebræ. Magendie has shewn the first rib to be as susceptible of motion as the other, provided its greater shortness be taken into account. It visibly rises at each inspiration. The inspiratory muscles tend to amplify the thorax in width, so that the direction backwards and upwards is always the preponderant; as is indeed manifest from the course of the muscular fibres. In any position of the body, provided the inspiratory muscles possess their due tone, they will effect this peripheral dilatation of the chest." (P. 44.)

Dr. Stromeyer points attention to the well-known circumstance that exciting passions (as joy) dilate, while those of an opposite description constrict the cavity of the chest, even to sense of suffocation; that during violent paroxysms of hysterical dyspnœa, the patient can draw a deep breath at will; proving, that neither the influence of volition, nor the action of the diaphragm or lungs is at fault; but that there is an interruption in the supply of nervous energy to the inspiratory muscles; and that, in like manner, public singers have suddenly lost their voice in certain complaints, and only recovered it after the use of gymnastic exercises. In such cases, it is not hoarseness but a want of power and intonation. There fails that resonance so essential to full vocal development, the result of a well expanded chest.

The author accounts as follows for the variation in the corporeal height between the morning and evening.

"Heretofore the diminution observed towards the close of day has been attributed to a compression of the intervertebral substance, which subsides with nocturnal repose. To expose the fallacy of such a mode of explanation, subject the lumbar spine to powerful pressure, taking measures at the same time to prevent lateral yielding, and no appreciable shortening will ensue. On the other hand, let this experiment be performed: Measure with exactitude the height of an individual standing upright; this done, bid him try to elongate himself without letting the soles of his feet quit the ground. Measure him now, and you will find him taller by a quarter of an inch or more. In the effort thus made, he merely diminishes the natural inflexion of the spine. The evening waning of the vital powers produces the opposite result." (P. 49.)

The next section is devoted to the consideration and (as Dr. S. believes) refutation of the currently received opinions concerning the causes of lateral incurvation, or Scoliosis. According to the author, writers on this subject inconsequently ascribe the deformity to an affection of the several muscles of the neck and trunk; while they contend, at the same time, that the constitutional derangements usually concomitant must operate on all the muscles.

"They did not take the pains to study individual muscles relatively to their function, so as to deduce therefrom the share each had in sustaining the healthy configuration of the body. Even skilful practitioners, as John Shaw for instance, peremptorily assert that the multiplied anatomical subdivisions of the dorsal muscles are so many integral parts of the same whole, conspiring to one and the same end. . . . Hence the consideration of curvature assumes quite another aspect, and acquires new interest, when directed to those particular muscles, the defective energy of which has first disturbed the general equilibrium. That the primary and secondary forms of incurvation are to be carefully discriminated, and that the organs of motion are not to be contemplated in a mass, is therefore obvious. . . . . To detect the peccant muscles, it is advisable, first of all, to select and set apart those of the trunk, which, from their modes of origin and insertion, cannot participate in the initial development of lateral incurvation. The latissimus dorsi, being distributed over the greater part of the back, its individual layers implicate but a small part of the spine; it can only, therefore, when acting partially, transform the dorsal and lumbar vertebræ into one large curve, the convexity of which would be towards the side of the impotent muscle. The longissimus dorsi and sacro-lumbalis are likewise insufficient to induce scoliosis; they influence less the lateral equipoise than the antagonism of the flexors of the trunk. . . . That paralysis of the longissimus dorsi and sacro-lumbalis produces incurvation anteriorly (Lordosis,) when double, and in addition lateral incurvation when single, has been tolerably well established. To the ordinary forms of lateral curvature, however, these deviations bear no similitude. . . . . The greater number of small fleshy bellies of the spinales and semi-spinales dorsi, multifidus spinæ, levatores costarum, interspinales, and intertransversales are also inadequate to produce curvature. They must be partially palsied on one side to determine that. Nor is it easy to conceive simultaneous paralysis of a number of muscles furnished with such different sets of nerves; yet, in order to illustrate the small serpentine curves of the spine, of which the arcs include but one or two vertebræ, it is needful to take the above muscles into account." (P. 51.)

That the abdominal muscles exert no real influence on the origin of lateral incurvation, may be inferred from the fact of their preserving their elasticity and tone, even under a high degree of this affection. Wherefore, after excluding so many muscles of the trunk, it will be seen that the few remaining are chiefly those concerned in the act of inspiration; and of these the author has instituted an elaborate analysis, of which we subjoin an abstract.

"1st. The sterno-cleido-mastoideus. Its influence on cervical incurvation has been long acknowledged, and by many intelligent writers held to be the only source. But the causes of that affect are manifold. That this muscle has no share in the origin of scoliosis cannot be reasonably imagined; indeed, the result of numerous cases prove the contrary. Where the head, for instance, acquires a bias, it is owing to the partial action of this muscle, so that it is turned in one direction, and the chin in an opposite. This is a condition not seldom met with, and which frequently is the only thing that awakens the attention of bystanders; the scoliosis meanwhile passing unheeded.

"2d. The trapezius, levator anguli scapulæ, and the rhomboidei. Of these muscles, the first is the one most commonly engaged, and that along with the mastoid; a circumstance plainly denoting the ground of its spasmodic or paralytic state to be referrible to the accessory, or nerve common to both. It sways the head principally, less the scapula. This is explainable upon the circumstance that the levator anguli scapulæ, and the rhomboidei, receive fewer branches of the above nerve; and thus the normal position of the shoulder-blade is preserved. Not long ago, I witnessed an instance of congenital paralysis of the left accessorius, in a delicate girl of three years old.

"3d. The scaleni. That these muscles contribute to the origin and development of scoliosis, we are led to conclude from those rare cases in which, together with considerable sinking-in of one side, the upper rib remains *in sitû*, and the shoulders exhibit a slight inequality in height. This state of things is tolerably constant in scoliosis induced by empyema, cured by evacuation outwards. The depression of the shoulder bears no relation to the sinking-in of the chest. . . . It is a matter of regret that Sir C. Bell has not directed his attention to the important function of the scaleni muscles. Concerning their respiratory nerves we know nothing. As they, however, receive branches of the cervical nerves from the third downwards, (Bock,) we may ultimately arrive at the truth, although difficult experimentally.

"4th. The serratus magnus. No muscle appears to me so much interested in the formation of scoliosis as this one. . . . . The lack of action of the inspiratory muscles in the so-called rhachitic curvature of the thorax, has not escaped observation. But the effect has been mistaken for the cause; inasmuch as it has been referred to the faulty direction of the ribs. For, should the ribs be depressed, they are nevertheless in a condition to be restored, provided the inspiratory muscles have sufficient power. On the other hand, if we suppose the impoverished energy of the serratus to be the cause, not the effect, of the phenomenon in question, we at once obtain a solution of all the symptoms. . . . The office of the serrati muscles is to keep the ribs stretched backwards, outwards, and somewhat upwards. Hence the diaphragm pulls the ribs inwards when the action of the serrati fails. This can be best seen during the tranquil respiration of delicate children. But the want of action is most conspicuous when we confine the movements of the diaphragm by firmly pressing on the belly, and thus bring the external inspiratory muscles into full play. Then the pectorales, trapezius, &c. act vigorously, whilst the serratus remains still; so that the capacity of the thorax is not augmented in breadth, but its sides are drawn inwards by the diaphragm. Again, on the subsidence of the action of the serratus, the diaphragm drags the ribs of the corresponding side inwards and downwards: hence the resulting concavity on one side of the chest, commencing immediately below the axilla. It is not always, at an early stage, associated with depression of the shoulder, because the upper digitations of the serratus pursuing a course from above downwards, the superior ribs are not necessarily dragged with it. . . . . The respiratory motions of the several inspiratory muscles of one side are so intimately united, that any imperfection in one is followed by a corresponding defect in the rest. For this reason, the impaired energy of the serratus must affect concurrently the trapezius. Hence the subsequent falling of the shoulder, the lateral deflection of the vertebræ; the longissimus being no longer able to sustain the lateral equilibrium. The disproportionate balance of the pectoral extremity now tends to increase the curve; yet this is of little moment, independently of other co-operative circumstances; such as the progressive affection of the inspiratory muscles, and the incidental consent of parts. This is evidenced from the fact that the symptoms do not get worse after the equipoise is lost, in the case of a contracted limb, or after empyema." (P. 59.)

Dr. Stromeyer thinks that, by referring the ordinary form of incurvation to one-sided paralysis of the serratus magnus, you are enabled to account for its most usual form, namely, that in which the thoracic vertebræ form one curve, and those of the neck and loins two others, for the purpose, as it were, of preserving the equilibrium. This *vis conservatrix* is the consequence of the involuntary efforts made by muscles still retaining their integrity of action; namely, the longissimus dorsi, sacro-lumbalis, and multifidus spinæ. It, moreover, coincides with the fact that healthy muscles gradually accommodate themselves to faulty positions of the skeleton, as is instanced in cases of unreduced luxation and ill-united fracture, where, in the course of time, the functions of the limb are partially restored; and, as is strikingly illustrated, in the very disease under consideration, in the dissections of Dr. Gunther, recorded in our last Number.*

The author has started some queries relative to thoracic rhachitis; as, whether the serrati are not always primarily affected, thereby furnishing one of the first elements of the serious constitutional malady in question, and one most likely to induce rapid destruction of the system, by its prejudicial influence on the function of respiration? or, in other words, whether chicken-breast does not, in every case, precede rhachitic incurvation? The frequent occurrence of chicken-breast without trace of diseased bony structure in the extremities, must at all events render it probable that the change in the form of the chest ought to be ascribed to something else than softening of the skeleton. This is a very important subject for further enquiry. If the question can be answered generally in the affirmative, and there be no accompanying softening of the skeleton, Dr. S. will have a strong argument in favour of his theory. In several cases which have lately come under our own notice, we found, on enquiry, that the chicken-breast was the first symptom that had attracted attention; and our former experience leads us to a like conclusion.

Dr. Stromeyer does not conceive the serratus magnus to be more liable to paralysis than any other muscle of inspiration; but contends its chief function to be respiratory. "The voluntary motion of the serratus appears highly problematical. After repeated examinations in muscular men, I am led to believe that, in the motions of the shoulder-blade, the serratus is only passive. We cannot rely on anatomists in such matters."

By pressure upon the abdomen, we impede the action of the diaphragm, and thus solicit the involuntary action of the external inspiratory muscles. In this manner we can, in every instance of lateral incurvation of the spine, demonstrate the paralysis of the serratus on the concave side of the curve. In voluntary deep inspiration, that muscle passively follows the ascension of the scapula. The author has experimentally proved the nervus thoracicus posterior to be its *energizer*, as conjectured by Bell. The intercostal muscles, according to Dr. Stromeyer, ensure a uniformly diffused action of the other inspiratory muscles over all the chest; more especially of those which, like the scaleni and sterno-mastoid, are affixed to small points remote from the inferior ribs. They are inadequate alone to keep up the dilatation of the chest. But those limited inflections of the ribs, sometimes found distinct from other curvatures, and situated near the sternum, he considers to be the effect of the disproportional action of these muscles. To their paralysis we must assign the collapse of the ribs observable in rickety subjects. The pectoral muscles, destined for preserving the arched form of the chest, when not counterbalanced by the action of the serrati, occasion chicken-breast. When deficient in energy, the thorax assumes a flat form, increasing in lateral diameter; and the diaphragm and triangularis sterni drag the sternum inwards. The supra-spinatus, and infra-spinatus, and subscapularis seem set apart for communicating tension to the pectoral muscles. The diaphragm participates in the formation of this disease, inasmuch as

* British and Foreign Medical Review, Vol. IV. p. 509.

it influences concurrently that portion of the chest, of which the external inspiratory muscles are enfeebled. The widening of the thorax in the axis of its long diameter, with simultaneous ascension of the abdominal viscera into its cavity, seems to be explicable only on the ground of diminished tone of that muscle.

The quadratus lumborum is principally engaged in upholding the action of the psoæ; it preserves the natural anterior inflection of the lumbar spine, and tends on both sides to sustain the balance of equilibrium. The author is in doubt whether its affections be not coexistent with those of the psoæ, or whether it be spontaneously capable of originating spinal disease. The psoæ, from their mode of insertion, are peculiarly adapted for preserving the corporeal equipoise; and, consequently, through partial paralysis of their involuntary power, for aiding in the development of curvature.

"The psoæ are extremely complex in their actions. Hitherto they have been regarded as flexors of the thigh, and of the upper part of the body when the thigh is fixed. They, however, are further intended to maintain the natural flexure of the lumbar vertebræ; not only necessary for the sake of equilibration, but likewise for relaxing the great expiratory muscles, and thus promoting the ascent of the chest during inspiration. In proof thereof, let a person, while standing voluntarily, depress the vertebræ of the loins, and attempt a full inspiration. Then will he experience, along the entire back, as far as the arm, the resistance offered by the latissimus and longissimus dorsi to the elevation of the ribs. Hence it is in a measure true that, proportionally as the back is bent with age, have the psoæ lost their organic tone, and is the breathing embarrassed. That these muscles can spontaneously determine these inflections of the lumbar vertebræ, is moreover established by the fact that the movement alluded to is not possible when the thighs are held closely approximated." (P. 86.)

The author has drawn some curious practical deductions from the study of this function of the psoæ, and endeavours to explain by its means the circumstance of the retraction inwards of the lumbar region during the acts of station and progression in congenital luxation of the hip-joint. He also explains, by its paralysis, the phenomena of what is commonly termed "the high hip;" but we cannot stop to notice the particulars.

In the fifth section of the work, the subject of permanent muscular contraction (*contractura*) is particularly discussed; a morbid condition which may be produced in one or other of the following ways:

"1st. By organic changes in the muscular texture itself, in consequence of inflammation, wounds with loss of substance, and the like.

"2d. By weakness and defective action in the antagonist muscles. This may proceed from—*a*. Wounds of the antagonist muscular bellies, or their tendons; *b*. Paralysis of the nerves of the antagonist muscles."

This paralysis may be complete or incomplete, and involve either the voluntary or the organic motions of a muscular part. Complete paralysis arises, for example, from division of a nerve, as the facial, producing contraction in the opposite muscles. Incomplete paralysis occurs, for instance, in the mastoid muscle. Here the influence of volition no longer serves to restore the bent head to its vertical position in many cases. It is further observable in certain people who involuntarily squint, but cease to do so when they direct attention to the circumstance. Here the paralysis of one muscle, which led to the contraction of its antagonists, is merely temporary, and removable at will. The motor nerves of volition are in a state of perfect integrity; those of organic movement are alone palsied.

"3d. By diminished influence of all the voluntary motor nerves of an extremity."

In reference to the prognosis of palsy generally, the presence of muscular contraction is a favorable indication; since the persisting tone and elasticity of the muscles prove the persistence of the *reflex-motory* power. Thus, as Dr. Stromeyer states, children labouring under paralysis of the lower extremities, the sequence of congenital hydrocephalus or hydrorachitis, generally recover the dormant functions after the tenth year of their age.

"4th. By the painful condition of a part; whereby its voluntary movements are crippled, as in articular inflammation. This resembles the preceding state, where the abstraction of the influence of the will is referrible to the nerves. The more powerful masses of flexor muscles equally preponderate over the extensors. The process of inflammation occurring in muscles, tendons, or the intervening spaces, often associated herewith, induces certain organic changes, which, after resolution of the inflammation in the joint, lead to contraction and false anchylosis.

"5th. By augmented power in a muscle. This is only possible during spasmodic action, where muscular motion is preternaturally increased; because increase of power is not compatible with prolonged repose. A muscle contracted for a length of time becomes progressively useless and atrophied." (P. 79.)

It is well known that, in a high degree of lateral incurvation, the face presents a peculiar relaxed look, and is often awry. The following observations on this condition are interesting:

"Medical practitioners regard this appearance as the sure criterion of a congenital and incurable disease. It is in reality not so. The distortion of the countenance is so far from being an unfavorable sign, that I have always known it to yield sooner than the curvature itself, when the respiratory energy of the muscles had been restored by judicious gymnastic treatment. Nay, the expression of the features visibly improves under the above plan, when the incurvation remains unaltered. . . . This phenomenon is intimately related with spinal curvature; and that side of the face uniformly wastes away whither the head inclines. . . . It is attributable to the interruption which the respiratory action of the cervical muscles has undergone. Their points of attachment being unduly approximated, they are incapacitated from exercising organic tension. Let it be understood that we here include not only the sterno-mastoid, but also the muscles of the upper part of the throat." (P. 105.)

The author adverts to a fact heretofore overlooked, namely, that the same predominant weakness of the left side usually recognized in curvature may be also present in the face; limited to its respiratory nerve, the facial: and, besides, "if we recollect the close union subsisting betwixt the brachial plexus and the nerves of inspiration, as the phrenic, thoracicus posterior, anterior, &c., we shall perceive that the less habitual use of the left arm must have a debilitating effect on the respiratory energy of that side, comprehending the neck and face."

According to Dr. Stromeyer, particular attitudes and postures of the body can have little, if any, control over the development of spinal incurvation. He, therefore, inveighs against the various contrivances,—such as straps, irons, and other *instruments of distress*,—which act by severe extension and pressure. These have been recommended by men whose skill in the application of mechanical principles has been generally commensurate with their ignorance of sound physiology; yet, however much importance he may attach to the efficiency of partial paralysis of the inspiratory muscles in determining lateral incurvation, he does not assign to it an exclusive prerogative.

"Far be it from me to undervalue the concurrence of other constitutional causes in the development of scoliosis; for I am convinced that a certain debility in the osseous system is required, in order that the irregular degree of stimulation, or (if we may use the phraseology) the incomplete and partial paralysis of the nerves of the respiratory muscles, may induce a permanent impression on the conformation of the body. Hence incurvation is a disease incident to the period of youth, rarely occurring in adult life. The perturbations to which the inspiratory muscles are obnoxious, after the skeleton has attained its mature growth, engender other affections,—as asthma, dyspnœa, and the like; which, transient or periodical in their character, cannot modify to any extent the compact bony framework. But this susceptibility to change of form, so remarkable in childhood, is not without its advantages. Thus, it exerts a sanatory influence in the event of external injuries, as falls, and in the case of empyema that has opened outwardly; recovery at that age being comparatively rapid, from the suppleness of the ribs. As respects the prompt formation of curvature, the degree of softness in the skeleton is very influential. Thus, in infants, that disease makes as much progress in the course of a few weeks as it will in as many years at a subsequent period of adolescence." (P. 114.)

Perhaps, one of the most frequent sources of paralysis of the muscles of inspiration is a long-continued cough, especially hooping-cough, in consequence of the oft-renewed powerful efforts made by these muscles when their antagonists are in a state of quiescence. That general rhachitis is also often associated with spinal curvature, and that there is frequently a transition of one disease into the other, (individuals, for example, who, in their early years have suffered from rickets, become towards puberty the subjects of incurvation,) are incontestable facts, and as such have been noticed by the author.

We shall conclude our analysis of Dr. Stromeyer's treatise with a brief abstract of his principles of treatment, referring to the original for fuller details.

"The theory that lateral incurvation is an affection of the respiratory nerves, unlike that proposed by Delpech, is in perfect accord with the commonly received therapeutic indications. . . . It is, indeed, interesting to reflect how much the powers of truth could, in the mind of an eminent observer, overbalance perverted pathological hypothesis; since, whilst Delpech attributed curvature, for the most part, to inflammation of the intervertebral substance, he recommended a mode of treatment diametrically opposed to his etiology of the disease. . . . The rational treatment of spinal curvature consists in improving the functions of assimilation and nutrition, and placing the body in such positions as shall be best fitted for animating the organic energy of the inspiratory muscles. These principles of cure suffice for the majority of cases; for the number is very small indeed in which the local disease is completely unconnected with constitutional causes. . . . The utility of gymnastic exercise is therefore proportional to its capability of meliorating the functions of assimilation and nutrition; and is in general so remarkable, that the exhibition of internal medicines is superseded, digestion being in a few weeks so much bettered, that the appetite is doubled, and all the functions restored to their healthy condition." (P. 131.)

Dr. Stromeyer has ascertained, in corroboration of his views, that well-directed gymnastic training communicates tone in an especial manner to the muscles of inspiration.

"It will be seen, in any orthopedic establishment, that not only do the muscles of the arm become larger and firmer, but the thorax often, in the course of a few months, gains several inches in girth. This is easily explained when we reflect that most gymnastic exercises are conjoined with suspension by the hands. Expansion of the chest naturally results from the increased vigour imparted to the inspiratory muscles; and manual suspension is the only means of ensuring the full action of the serratus magnus, as it compels it to support part of the bodily weight. Sometimes, it is true, in inveterate scoliosis, many months may elapse ere the serratus of the concave side of the curve will resume its activity; although, while the patient is hanging by the hands, the concavity disappears from the elevation of the ribs. It is absurd to believe that such exercises can only avail when the inflexion is situated below the level of the shoulder; because, as paralysis of the serratus becomes sooner or later coupled with atony of the trapezius, the scaleni, and the remaining muscles of inspiration, restoration of its functions will actuate consentaneously these others. . . . Diversified exercises, performed in the company of others, are alone advantageous; partly because a considerable time may be so devoted to them, and partly because, when so conducted, they cease to be irksome, and young females find them really pleasurable. Balance of power and bilateral symmetry are the result." (P. 133.)

The author countenances, under certain limitations, the employment of gentle mechanical means,

"On the one hand, because the secondary phenomena of scoliosis, as the shortening of particular muscles, stiffness and rigidity in the costal and vertebral articulations, require the intervention of mechanical extension, so as to fit these again for service; on the other, because moderate extension, especially of an elastic kind, acts as a stimulant to the lax muscular fibre. In proof of this, we need only look to the augmented bulk the calf of the leg acquires when club-foot is treated by extensional apparatus." (P. 135.)

He recommends the *stretching-bed*, as he terms it; and chiefly for the reason that, when the patient is placed either horizontally or upon an inclined plane, a very slight spring-force suffices for maintaining the adequate pitch of tension, inasmuch as the resistance from the weight of the body is then null. He is very far, however, from countenancing that extreme degree of mechanical extension, which, when combined, as it usually is, with almost total inactivity of the body, we see productive of such lamentable results in the practice of some persons in this country.

"It is a vulgar and a dangerous mistake to suppose that, by powerful apparatus and forcible traction, you can speedily subdue spinal incurvation. Undoubtedly, if you stretch the patient on his back, you straighten the spine; but, by repetition of this racking process, his general health becomes impaired, his muscles enfeebled, his cartilages loosened, and, upon his resuming the vertical position, the original distortion is again present, and sometimes in a more aggravated form than before." (P. 136.)

The method of treatment where confinement to the lateral position is advocated, by which the superior half of the chest is elevated, and its external inspiratory muscles brought into full activity, leads the author to comment upon the views of MM. Pravaz and Guérin, who have adopted this judicious practice, despite their false reasoning concerning the causes of curvature.

"The patients are obliged to lie in a kind of hammock, upon the convex side of the curve; of which side, by different movements, the energy of the muscles is meanwhile quickened. Future experience can only decide on the value of this plan; at all events the posture is comfortable. . . . It is desirable to conjoin some sort of mental occupation with it. Of course, it is improper for those cases in which the palsy of the inspiratory muscles is not strictly one-sided: for these the supine position is decidedly preferable, as a slight degree of extension can be advantageously conjoined, and free play allowed to both lungs." (P. 137.)

Dr. Stromeyer conceives the views he has unfolded will materially facilitate the early diagnosis of such affections.

"By studying attentively the external inspiratory motions, we shall not only be enabled to distinguish, with precision, slight curves, but likewise to predict and counteract their supervention. Since paralysis of the inspiratory muscles does not immediately produce change of form, a considerable interval may elapse before the latter criterion is appreciable. Therefore, the possibility of preventing distortion by simple measures at the commencement ought to awaken public attention on this head."

For the purpose of investigating the respiratory movements of the external muscles of inspiration, we ought, as formerly mentioned, to make pressure upon the abdomen, according to the mode of examination introduced by Bichat, in reference to another object.

"Delicate children are best suited for learning this plan at first. As with them the want of atmospheric air is great, thoracic respiration is produced so soon as the abdomen is compressed; and, in consequence of the extreme mobility of the joints and cartilages of their ribs, the motions of the latter are more extensive and more palpable than in older subjects. Hence, in them, it is easy to trace the defective elevation and imperfect lateral expansion of the concave side of the chest, depending on scoliosis. The reason why palsy of the serratus muscle cannot be detected in voluntary deep inspiration, both sides of the thorax rising equably, may be understood when we reflect that the diaphragm, by distending the lungs with air and compressing the abdominal viscera, facilitates the elevation of the thorax. Then, only a slight consentaneous effort of the inspirators is requisite; whereas, on compressing the abdomen, the motion of the diaphragm is constrained, so that the task of filling the lungs devolves upon the external inspiratory muscles, which those of the concave side are no longer competent to fulfil. In a large amount of cases of scoliosis, in individuals up to the age of eighteen years, recently examined by me, I have found this relation constant: it failed, on the other hand, in those instances where an amendment had been previously wrought by a course of gymnastic exercise." (P. 141.)

The various topical remedies which seem to act by stimulating the nerves of the part, promoting the circulation of blood, and consequently favoring the increase of muscular size and strength,—as friction, shampooing, and the like,—ought, according to the author, to be applied to the concave, and not the convex side, as commonly prescribed.

"Of the beneficial effects of stimulant liniments (Liq. Ammoniæ ℥j. c. Sp. Vini rect. ℥vij.) to the region of the serratus muscle, we have ample evidence from the pressio abdominalis test, tried before and after. That such effects will not be lasting unless the function of nutrition be at the same time exalted, may be reasonably inferred. Therefore, no great reliance is to be placed on the use of external treatment alone. In the instance of *sinuous* incurvation, the stimulant frictions are to be made over all the outward muscles of inspiration." (P. 143.)

In concluding this brief notice of Dr. Stromeyer's book, we must remark, that its practical value is likely to be diminished by the too rigid adherence of the author to the theory by which he wishes to explain all the phenomena of the disease. As we said at the beginning of the article, we even doubt the truth of the theory altogether, if, by the term *paralysis*, as here applied, we are to understand that kind of loss of muscular power which is usually termed paralysis in this country. If he will allow us to read *debility* for *paralysis*, muscular debility produced by muscular inaction, we shall not differ very widely from him in many of his views. It is certainly true that, while the author has rendered a positive service, by forcibly calling attention to the influence of *inaction* of the inspiratory muscles in weakening the natural supports of the spine, and thus contributed to improve our means of both prevention and cure, he is disposed to make his fundamental proposition much more general and sweeping than strict observation would warrant, and to deduce many of his peculiar views from a theoretical enlargement and one-sided consideration of his facts, rather than from their plain and literal meaning. In many instances he traces the absence of respiratory motion to nervous paralysis, where other observers would ascribe it to muscular debility dependent solely on inaction. Experience does not warrant the belief of real paralysis being so common as his doctrine would make it; and why should it lay hold of the inspiratory nerves, in preference to all others? If, on the other hand, the evil is debility from inaction, we can at once understand the history, symptoms, and progress of the case. We cannot see any good *a priori* reason why paralysis of the accessorius should so often attack and lead to curvature in females, and so rarely in males; but we see a very cogent reason why muscular debility should arise from muscular inaction in quiescent and corset-encased females, who cannot distend their chest even to save their lives,—and why boys and active and uncompressed girls should escape. That nervous debility accompanies muscular is no doubt true; but that is quite a different thing from paralysis, and owns a different cause. We could point out females whose muscles employed in deep inspiration, have not moved a fibre for months, or perhaps years; not from paralysis of their motor nerves, however, but from sitting all day working in constrained or bent positions, and enclosed in stays which prevent any considerable expansion of the ribs. In many such cases, we believe that the power of making a deep inspiratory movement is lost for the time by disuse, but requires only freedom, and the natural action of the inspiratory muscles, to be restored.

That an inactive and debilitated state of the inspiratory muscles conduces to spinal curvature, we fully believe, because their healthy action is evidently conducive to a firm and erect carriage; and Stromeyer deserves credit for shewing the share they have in the result, and its *modus operandi.* The indifferent general health, common in such cases, seems to us to be produced not by the curvature, but by the previous mode of life impeding an important function—respiration, and debarring the exercise without which health can scarcely be looked for. It is aggravated by, and is the concomitant, but neither the cause nor the effect of the curvature.

We quite agree with Dr. Stromeyer, that numerous cases of short and difficult breathing are owing more to want of muscular action than to the state of the thoracic contents themselves; and to the improved action of the inspiratory muscles we have been accustomed to ascribe the freer breathing which many invalids, suffering from chronic diseases of the chest, experience after exercising moderately for a few weeks on hilly ground. In such cases, a part of the benefit is no doubt owing to a less congested state of the lungs and internal viscera, but much is owing to more vigorous inspiration. In the improvement of the wind in training, we have no doubt that a great deal arises also from increased tone and readiness in the inspiratory muscles. The good effects of lecturing and reading aloud are partly owing to the same cause. Hence the evil of the quiescence creeping into the modern amusements of the young. Many girls, and even some boys, scarcely know what a full deep inspiration is, and hence their chests never become adequately expanded, or the trunk vigorous and erect. When the deep inspiratory muscles are not adequately developed by habitual exercise, the shoulders fall down by their mere weight, diminish the caliber of the chest, and act as weights against its expansion. It may be that, in some instances, the primary cause is want of nervous influence to the inspiratory muscles; but we much doubt if it is often so, except from the patients being deprived of such plays, pastimes, and exercises as constitute nervous stimuli. It is no loss of power inherent or originating in the nerve. We have often observed benefit from regular friction along the cervical and upper dorsal spine, and over the shoulders and scapula; but we regard that fact in the same light, first, as in itself exercise to the very muscles which want it, and, secondly, as promoting their nutrition and vigour, by the friction acting directly on their vessels and nerves. Of course, the spinal nerves are stimulated, and aid in the result, but still not as in real paralysis.

